# Characterization of Fluorescent Eye Markers for Mammalian Transgenic Studies

**DOI:** 10.1371/journal.pone.0029486

**Published:** 2011-12-28

**Authors:** Jonathan C. Cornett, Sean F. Landrette, Tian Xu

**Affiliations:** 1 Department of Genetics, Boyer Center for Molecular Medicine, Yale University School of Medicine, Howard Hughes Medical Institute, New Haven, Connecticut, United States of America; 2 Institute of Developmental Biology and Molecular Medicine, Fudan-Yale Center for Biomedical Research, School of Life Sciences, Fudan University, Shanghai, China; The University of Sydney, Australia

## Abstract

Genotyping mice by DNA based methods is both laborious and costly. As an alternative, we systematically examined fluorescent proteins expressed in the lens as transgenic markers for mice. A set of eye markers has been selected such that double and triple transgenic animals can be visually identified and that fluorescence intensity in the eyes can be used to distinguish heterozygous from homozygous mice. Taken together, these eye markers dramatically reduce the time and cost of genotyping transgenics and empower analysis of genetic interaction.

## Introduction

Transgenic and knockout studies in mice and other mammals are critical in understanding gene function as well as modeling human diseases. However, isolation of genomic DNA and identification of genetically modified animals using Southern blotting or PCR-based genotyping can be costly and time consuming especially when crosses involve multiple genetic alterations. Visible transgenic markers, such as eye color and fluorescent protein markers, are commonly used in invertebrate and some vertebrate model systems to identify transgenic animals [1], [2], [3]. Although ubiquitous fluorescent proteins have been used successfully in mice to label tissues and cells [4], [5], their utility as transgenic markers is limited as they can interfere with studies using fluorescent protein fusions or lineage markers [6], [7]. A more efficient marker system to facilitate genotyping and reduce animal costs is extremely desirable.

## Results

**Figure 1:**
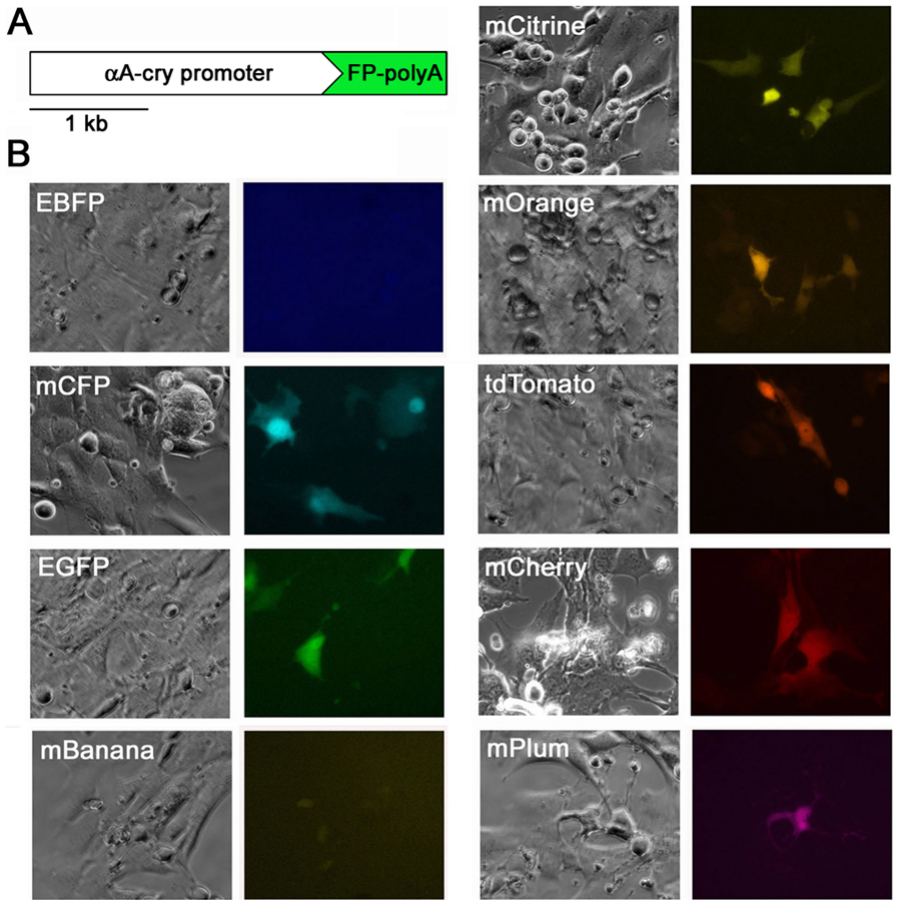
Expression of αA-crystallin driven fluorescent proteins in α-TN4 mouse lens epithelial cells. (A) Schematic map of αA-crystallin fluorescent protein constructs drawn to scale. Features include the 2.5 kb αA-crystallin promoter and the 1 kb fluorescent protein coding sequence and polyadenylation signal (1.6 kb for tdTomato). (B) Fluorescent protein expression 48 hours post-transfection. Images were taken in grayscale and pseudo color added using AxioVision digital image processing software.

**Figure 2:**
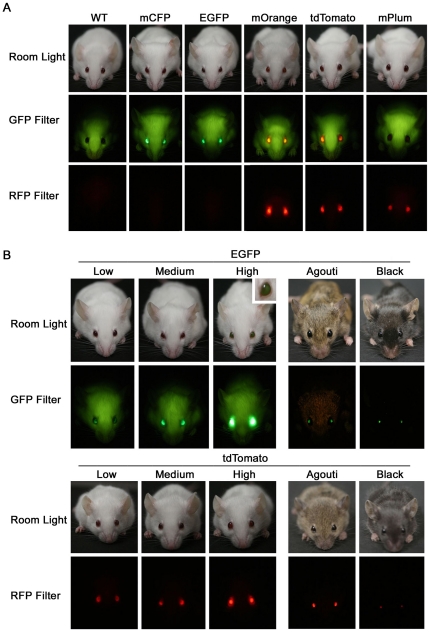
Fluorescent eye marker visibility and variability. (A) Fluorescent proteins expressed in the lens can be visualized under a handheld flashlight. All five fluorescent eye markers can be seen using the GFP flashlight while only mOrange, tdTomato, and mPlum can be seen using the RFP flashlight. (B) EGFP and tdTomato marker expression varies between transgenic lines (Low, Medium, High). Inset shows EGFP “high” line is visible in room light with no excitation. EGFP “medium” and tdTomato “high” transgenic lines are also shown in pigmented (Agouti, Black) backgrounds. Note that the visibility of fluorescent eye markers is reduced in pigmented backgrounds.

We sought to develop a set of fluorescent protein markers that could be easily employed and widely applicable for genetic studies in mice and other mammals. We generated marker constructs containing nine different fluorescent proteins ranging from blue to far-red spectral emissions [8] under the control of the mouse αA-crystallin promoter which is highly expressed specifically in lens epithelial cells [9]. To test these fluorescent eye markers, we transfected them into mouse lens epithelial α-TN4 cells [10] (Fig. 1) and visualized them by fluorescent microscopy. Based on overall brightness and spectral separation, five marker proteins (mCFP, EGFP, mOrange, tdTomato and mPlum) [11], [12], [13] were chosen for *in vivo* testing in transgenic mice. We generated transgenic mice by pronuclear injection of fluorescent eye marker DNA with unrelated transgenes (see Methods). The unrelated transgenes included *piggyBac* (PB) transposon mutator constructs and PB transposase (*PBase* or *PBaseER*) constructs for somatic forward genetic screens [14]. It has been previously reported that when two linear DNA fragments are co-injected in the generation of transgenic mice, they often co-integrate into the genome as a transgene concatamer containing both transgenes [5], [15], [16], [17], [18]. Thus, when present in the same transgene concatamer, the fluorescent eye marker can reliably indicate the presence of the unrelated transgene. A total of 32 founder mice carrying the fluorescent eye markers were selected after PCR genotyping and backcrossed to the FVB/NJ background. All of these founders transmitted the eye marker to their offspring. To determine the visibility of fluorescent proteins in the eye, we examined transgenic mice under a portable dual fluorescent flashlight. Twenty-seven lines out of the 32 (84%) showed visible fluorescence after excitation under the portable flashlight (Fig. 2A). These eye markers were also visible under a portable longwave UV lamp as described previously with ubiquitous fluorescent markers [4], [5]. Fluorescence intensity varied between different lines with the dimmest being visible only after excitation in a dark room and the brightest lines being visible in room light even without excitation (Fig. 2B). We also crossed some lines onto black and agouti backgrounds to analyze how pigment affects visualization of the fluorescent eye markers. The presence of pigment reduced the intensity of fluorescence (Fig. 2B). Nevertheless, the high and medium EGFP and tdTomato lines are still easily visible in both agouti and black backgrounds even when the room light is on. However, the low expressing reporter lines are faint in pigmented mice, although they are still detectable in a dark room. Thus, these reporters can be used for both pigmented and non-pigmented mice. Importantly, we have maintained these lines over many generations (>25 generations in some lines) and we have not observed inactivation of the fluorescent markers over time. Thus eye marker fluorescence can be consistently followed from one generation to the next.

**Figure 3:**
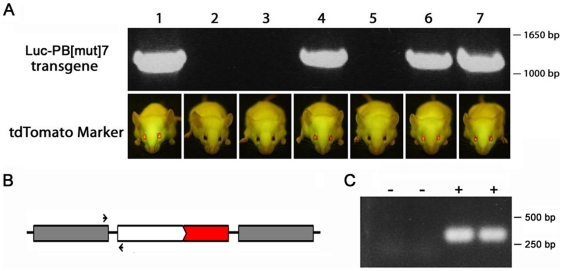
Co-segregation and co-integration of fluorescent eye marker and transgene. (A) tdTomato fluorescent eye marker co-segregates with and accurately marks the presence of an unrelated transgene (*Luc-PB[mut]7*). One litter of pups numbered 1-7 is shown. The unrelated transgene was amplified by PCR (upper panel) and the tdTomato marker was visualized under the GFP flashlight in room light (lower panel). (B) Schematic drawing of PCR strategy. One PCR primer (small arrows) was designed against the 3′ end of the *Luc-PB[mut]7* transgene (gray box) and the other primer against the αA-crystallin promoter (white box) driving the tdTomato marker transgene. (C) The hybrid PCR product from the transgene concatamer is present in two transgenic animals (+) but not wildtype littermates (−).

We next addressed the question of whether the eye markers could be used to reliably identify the presence of the unrelated transgene with which they were co-injected. Of the 27 transgenic founders with visible fluorescent eye markers, 21 (78%) were shown by PCR genotyping to also carry the unrelated transgene. To establish co-integration of the eye markers and the unrelated transgene, we studied their segregation in the F_1_ and subsequent generations. Animals with visible fluorescence in the eye, as well as littermates with no fluorescence, were analyzed by PCR for the presence of the unrelated transgene. It was assumed that co-integration at a single site occurred if all animals with visible fluorescence in the eye were positive for the unrelated transgene while all animals with no fluorescence in the eye were negative for the unrelated transgene. Of the 21 lines analyzed, 20 (95%) showed co-segregation of the transgene and its eye marker (for example see Fig. 3A). In all of the transgenic lines where co-segregation was observed, the transgenes have segregated together in all subsequent crosses (>200) indicating that mice can be efficiently genotyped for the unrelated transgene by screening for the presence of the fluorescent eye marker. In one instance the transgene segregated from the marker in the F_1_ generation. Thus, in some situations, it may be beneficial to combine the two onto a single construct. To further confirm co-integration of both transgenes into a single genomic site, we performed PCR analysis with primers designed to amplify the region between the eye marker and its neighboring transgene. In all five transgenic lines tested, the PCR product was present in transgenic animals but not wildtype littermates (Fig. 3B and Fig. S1). These results confirm that the fluorescent eye markers co-integrate with the unrelated transgenes into the same transgene concatamer.

To determine if the fluorescent eye markers affect the expression patterns of the co-injected transgene, we performed immunofluorescent staining in several lines where the EGFP marker was co-injected with a conditional transposase (PBaseER) for the PB transposon. PBaseER expression in these lines was restricted as expected to the epidermis and hair follicle by the human keratin 14 promoter [19] or the mouse P-cadherin promoter [20] (Fig. S2). When crossed with mice carrying a mutagenic piggyBac transposon, the double transgenic animals develop skin tumors providing further evidence that the expression and function of the co-injected transgenes are not affected by the eye markers [14]. Importantly, we did not observe ectopic expression of the EGFP fluorescent eye markers in the skin in these lines (Fig. S2). Thus, the fluorescent eye markers are specifically expressed and do not affect the proper expression and behavior of the co-injected transgenes.

**Figure 4:**
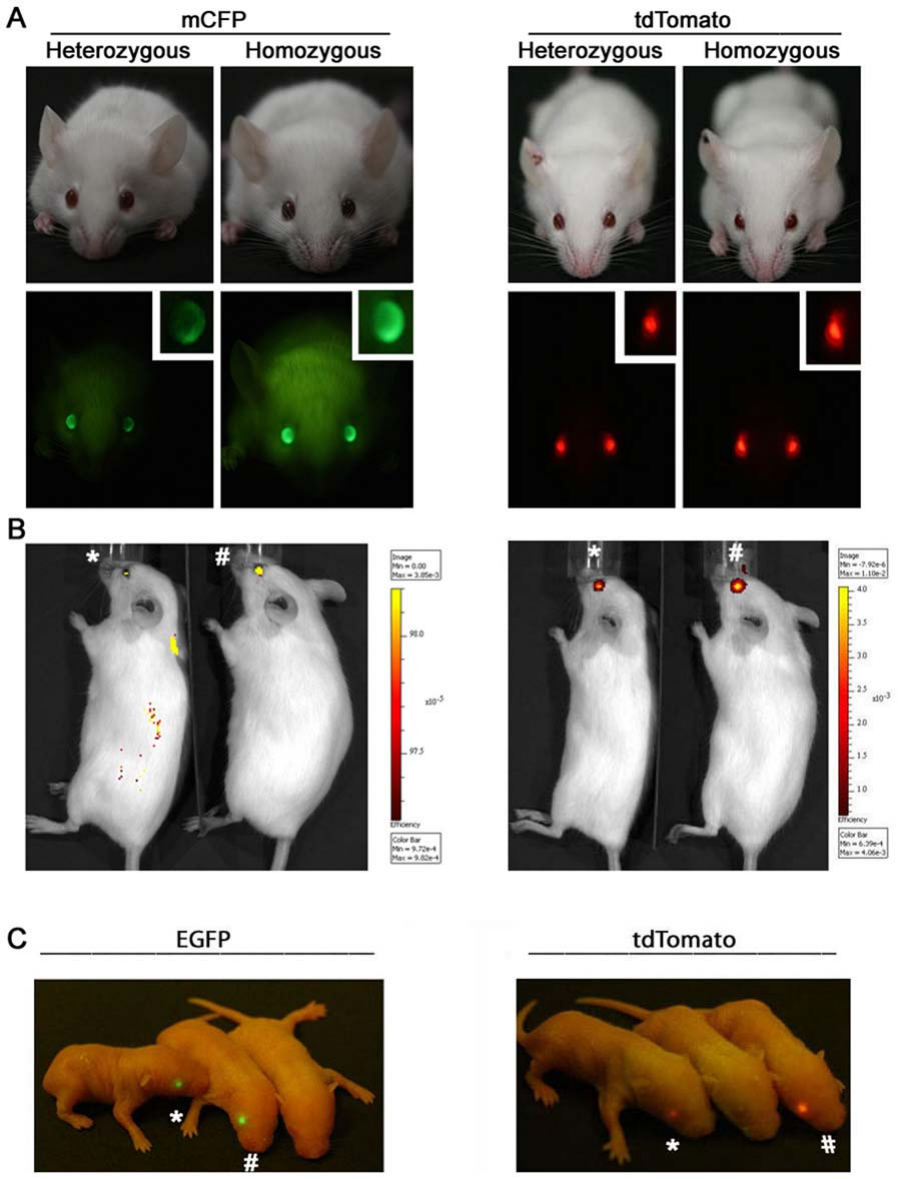
The intensity of the fluorescent eye markers allows differentiation of heterozygous from homozygous transgenic mice. (A) Heterozygous and homozygous adult mice carrying mCFP or tdTomato markers can be easily identified under a handheld flashlight. (B) Homozygosity in adult mice can also be determined more quantitatively using whole animal *in vivo* fluorescence imaging. (C) Neonatal mice carrying EGFP or tdTomato markers can also be easily identified as heterozygous or homozygous under a handheld flashlight. (* marks heterozygous animals, # marks homozygous animals)

Next we asked if fluorescent intensity of the eye markers correlates with gene dosage. We self-crossed mice that were heterozygous for mCFP, tdTomato or EGFP eye markers and analyzed marker fluorescent intensity in their offspring. Both adult (Fig. 4A) and neonatal (Fig. 4C) offspring could be separated as heterozygous or homozygous for the marker by visualizing fluorescence intensity under a portable flashlight. We could also use whole animal *in vivo* fluorescence microscopy to identify homozygous from heterozygous transgenic mice (Fig. 4B). When these homozygous animals were crossed to wildtype FVB/NJ mice, all offspring carried the marker transgene as expected. Ketamine-xylazine anesthesia is frequently used in animal manipulations and can cause acute reversible cataracts in mice [21]. We found that marker fluorescence intensity increased with acute cataract formation (Fig. S3). Taken together, these fluorescent eye markers make it possible to quickly and accurately identify homozygous transgenic mice.

**Figure 5:**
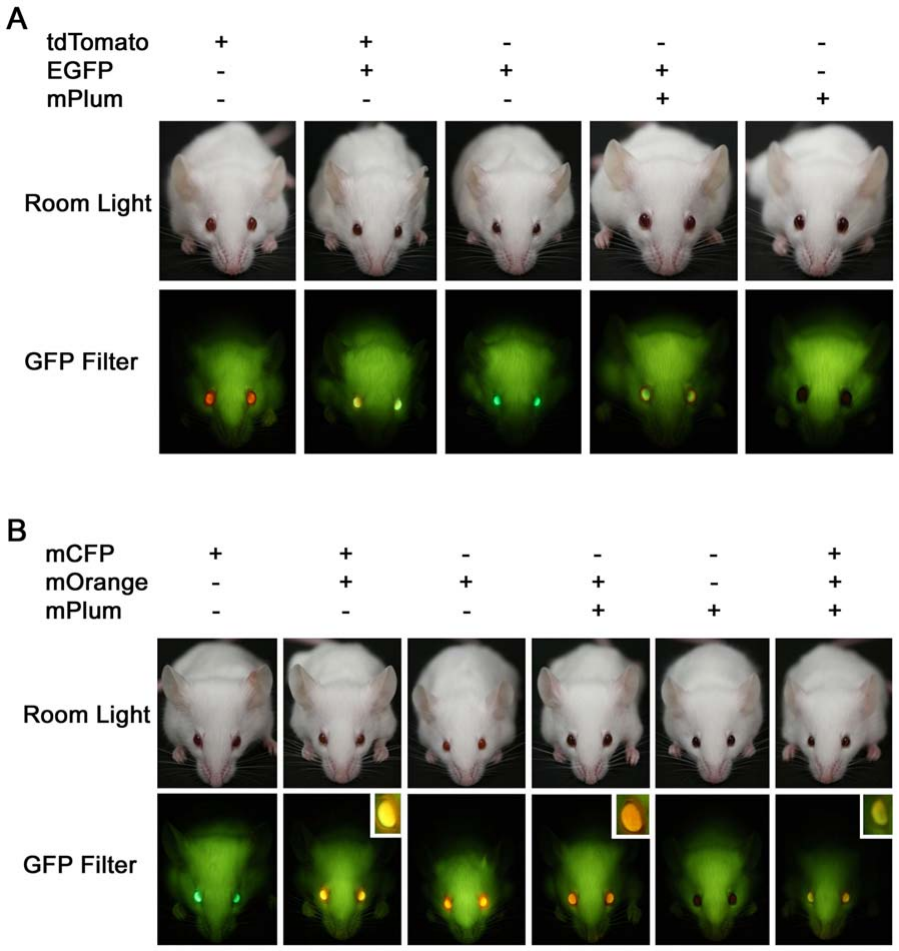
Fluorescent eye markers allow visual identification of double and triple transgenic mice. (A) Double transgenic mice carrying the EGFP marker and either tdTomato or mPlum can be easily distinguished from single transgenic littermates under the GFP flashlight. (B) Single, double and triple transgenic mice can be identified using mCFP, mOrange and mPlum eye markers. All six possible combinations are distinguishable under the GFP flashlight. Insets show close up view of fluorescence in the eyes of double and triple transgenic mice.

Finally, we asked if the fluorescent eye markers could be used to distinguish mice that carry more than one transgene. To address this question, we sat up matings between transgenic mice with different color eye markers. We evaluated eight different two marker combinations in all. Indeed, in seven of the eight combinations, double transgenic mice were easily distinguished from mice carrying a single transgene by visualizing fluorescent markers in the eye (Fig. 5 and Table S1). For example, EGFP and tdTomato double transgenic animals could be distinguished by a yellow eye color while the single transgenic eyes appear green and red respectively (Fig. 5A). We also found that the triple marker combination of mCFP, mOrange and mPlum could be used to identify and differentiate triple transgenic animals (Fig. 5B). Thus, the different fluorescent eye markers can be used to distinguish mice carrying up to three different transgenes without the need for PCR genotyping.

## Discussion

In summary, we developed and characterized a set of broadly useful fluorescent eye markers that allows for the rapid and easy detection of transgenic mice. We demonstrated that these eye markers can be used to visibly follow the segregation of transgenes from one generation to the next using a handheld flashlight. This method is non-invasive and eliminates the need to subject transgenic animals to the stress of isolating tissue for genomic DNA extraction and genotyping. Moreover, visual identification of eye markers can be performed from postnatal day one to adult while tissue sample removal is limited to a short window of time. In addition, we showed that marker fluorescent intensity correlates to transgene dosage allowing for quick identification of homozygous transgenic animals. We were also able to easily distinguish single, double and triple transgenic mice by marking different transgenes with unique color eye markers. Analysis of animals with multiple transgenes or genetic alterations is routinely done in invertebrate model organisms but has been difficult for mammals. The ability to differentiate mice with multiple genetic alterations will greatly facilitate analysis of epistasis and other genetic interactions. Although we limited our analysis to transgenic animals in the present study, these fluorescent eye markers could potentially be incorporated into gene targeting constructs for knockout studies. In addition, as the crystallin promoter is highly conserved in vertebrates, these eye markers could also be used for genetic studies in other mammalian model systems. The fluorescent eye marker system described here is widely applicable for mammalian transgenic work, not only reducing both the resources and the time traditionally required, but also further empowering genetic analysis.

## Materials and Methods

### Ethics Statement

All experiments were approved by and conducted in compliance with the Yale Animal Resources Center and the Institutional Animal Care and Use Committee under protocol number 2008-10230.

### Construction of transgenic constructs

The αA-crystallin-EGFP plasmid, pA425, was described previously [9]. Coding sequences for EBFP (Clonetech), mCFP[12] and mPlum[13] were PCR amplified using primers that contained a BamHI recognition site on the 5′ and EcoRI recognition site on the 3′. All other fluorescent protein coding sequences were released from the pRSET-B vector by digestion with BamHI and EcoRI. All fluorescent protein coding sequences were then cloned into the BamHI/EcoRI sites in pEF6/V5-His (Invitrogen). Finally, they were released from pEF6/V5-His by BamHI/NotI digestion and cloned into pA425 to replace the EGFP coding sequence. All fluorescent marker constructs will be available upon request.

### Cell Culture, transfections and imaging

Mouse lens epithelial α-TN4 cells were cultured in Dulbecco's modified Eagle's medium containing 10% fetal bovine serum, 100 µg/ml penicillin and 100 µg/ml streptomycin in a humidified 5% CO_2_ atmosphere. Cells in 24-well plates were transfected with 0.8 µg plasmid DNA using 2 µg Lipofectamine 2000 (Invitrogen). After 48 hr. cells were imaged using the Axio Observer.A1 inverted fluorescent microscope (Carl Zeiss).

### Generation of transgenic mice

All transgenic mice were generated at the Yale University Transgenic Mouse Core Facility. Transgenic mice were produced by microinjecting the purified MluI and AflII fragment of αA-crystallin marker constructs with linearized unrelated transgenes at a ratio of 1∶3 into FVB/NJ or (C57BL/6J X SJL/J) F2 fertilized eggs. Transgenic founders were identified by PCR genotyping of genomic DNA with forward primer A-CRY (5′-GCTCCTGTCTGACTCACTGC-3′) and reverse primers FP-R2 (5′-GGAATTCTTACTTGTACAGCTCGTCCATG-3′) or MPLUM-R (5′-CGGAATTCTTAGGCGCCGGTGGAGTGG-3′) for αAcry-mPlum. Founders were mated with FVB/NJ mice to establish transgenic lines.

### PCR

Luc-PB[mut]7 transgenic mice were genotyped by PCR genotyping of genomic DNA with forward primer LucPBLF (5′ TGAATACGATTTTGTGCCAG 3′) and reverse primer LucR (5′ GGATCCTTATCGATTTTACC 3′) yielding the expected product size of 1.4 kb. For detection of hybrid PCR products spanning the junctions between the eye markers and transgenes of interest in transgenic concatamers, the following primers were used: PBase3′-F (5′ ACATATGGGAGGGCAAATCA 3′), Cry promoter-R (5′ AGCCTGGAAGTAGACCAGCA 3′), EGFP pA-R (5′ CCCCCTGAACCTGAAACATA 3′), Pcad Promoter-R (5′ TCTGGCACCCCCAATATAAA 3′), and Luc3′-F1 (5′ CGTCGCCAGTCAAGTAACAA 3′).

### *In vivo* imaging of fluorescent eye markers

For fluorescent photographs, mice were anesthetized using ketamine/xylazine (100mg/kg, 10mg/kg) and marker fluorescence was excited either in room light or a dark room using the DFP-1 Dual Fluorescent Protein flashlight (Nightsea). Photographs of fluorescent eye markers were taken using an Eos Rebel XTi digital camera (Canon) using the following exposure settings: ISO 1600, 1/100 second, f 5.6. The camera was fitted with appropriate 58mm lens filters to block the emission light. For GFP we used the BB58 barrier filter (Nightsea) and for RFP we used a Red 25 filter (Tiffen). For *in vivo* fluorescent microscopy, mice were anesthetized with isoflurane using the XGI-8 gas anesthesia system and imaged using an IVIS Spectrum (Xenogen Corporation-Caliper Life Sciences). Epi-fluorescent images were taken using different combinations of filter sets and light outputs were quantified using Living Image software (Caliper Life Sciences). Images were processed using Adobe Photoshop software.

### Immunofluorescence of frozen skin sections

Mouse backskin was immediately embedded in OCT, frozen, and sectioned. Sections were fixed for 10 min in 4% PFA in PBS, washed three times for 5 min in PBS, and permeabilized in 0.1% Triton for 15 minutes at RT. Sections were then washed three times for 5 min in PBS and blocked for 1 hour at RT in a humidified chamber using the following blocking solution: 5% NGS, 1% BSA in 0.1% Triton X in PBS. Primary antibody staining against the estrogen receptor (ERα) was done overnight at 4^o^ using rabbit polyclonal ERα anitibody (MC-20:sc-542,1∶100, Santa Cruz Biotechnology). DAPI (1∶3000) was used for nuclear staining. Stained slides were imaged using the Axio Observer.A1 inverted fluorescent microscope (Carl Zeiss).

## Supporting Information

Figure S1 Co-integration of fluorescent eye markers with PBaseER transgenes. (A) Schematic drawing of PCR strategy for K14-PBaseER and Act-PBaseER transgenic lines (on left). One PCR primer (small arrows) was designed against the 3′ end of the *PBaseER* transgene (gray box) and the other primer against the 3′ end of the EGFP marker transgene (green box). The hybrid PCR product from the transgene concatamer is present in transgenic animals (+) but not wildtype littermates (-). (B) Schematic drawing of PCR strategy for LSL-PBaseER and Pcad-PBaseER transgenic lines (on left). One PCR primer (small arrows) was designed against the 3′ end of the *PBaseER* transgene (gray box) and the other primer against the αA-crystallin promoter (white box) driving the EGFP marker transgene (white box). The hybrid PCR product from the transgene concatamer is present in transgenic animals (+) but not wildtype littermates (-).
(TIF)

Figure S2 Expression of co-injected transgenes is not affected by fluorescent eye markers. Immunofluorescent staining of frozen skin sections from **(A)** WT, **(B)** K14-PBaseER (3 independent lines), and **(C)** Pcad-PBaseER (1 line) shows expression as expected in the epidermis and hair follicles. Note that ectopic fluorescence from the eye markers was not observed.
(TIF)

Figure S3 Anesthesia-induced acute cataracts affect fluorescent eye marker intensity. (A) Heterozygous and homozygous adult mice carrying tdTomato markers can be easily identified under a handheld flashlight. Mice imaged quickly have not developed cataracts. (B) tdTomato marker intensity increases following acute cataract formation (inset). Mice were imaged following ten minutes of anesthesia.
(TIF)

Table S1 Fluorescent eye marker combinations.
(DOC)
