# Supplementary figures and images for: Characterization of Fluorescent Eye Markers for Mammalian Transgenic Studies
Source: PLoS One. 2011 Dec 28;6(12):e29486. doi: 10.1371/journal.pone.0029486 (PMC3247282; doi:10.1371/journal.pone.0029486)

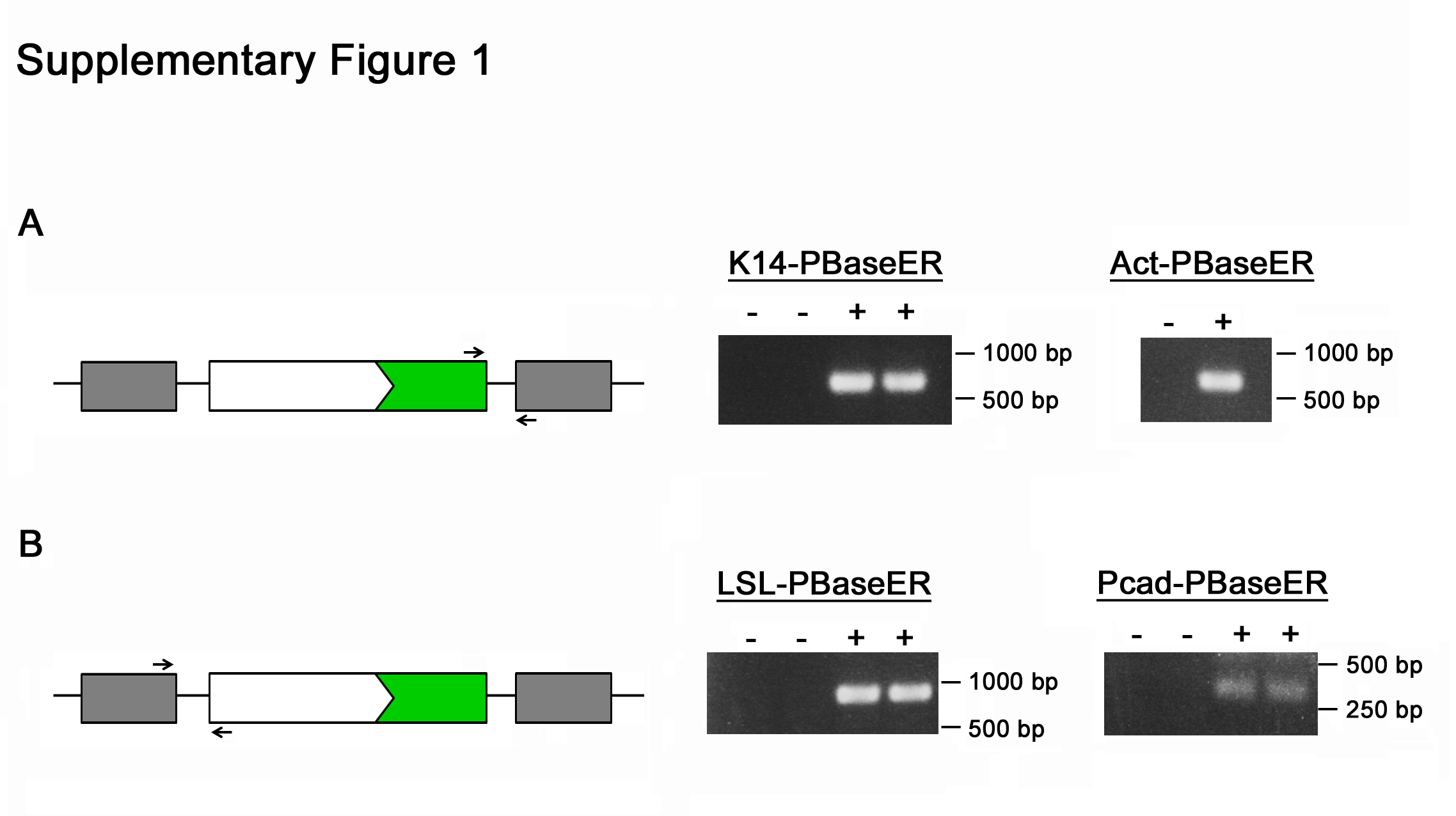

Supplement: Figure S1 — Co-integration of fluorescent eye markers with PBaseER transgenes. (A) Schematic drawing of PCR strategy for K14-PBaseER and Act-PBaseER transgenic lines (on left). One PCR primer (small arrows) was designed against the 3′ end of the PBaseER transgene (gray box) and the other primer against the 3′ end of the EGFP marker transgene (green box). The hybrid PCR product from the transgene concatamer is present in transgenic animals (+) but not wildtype littermates (-). (B) Schematic drawing of PCR strategy for LSL-PBaseER and Pcad-PBaseER transgenic lines (on left). One PCR primer (small arrows) was designed against the 3′ end of the PBaseER transgene (gray box) and the other primer against the αA-crystallin promoter (white box) driving the EGFP marker transgene (white box). The hybrid PCR product from the transgene concatamer is present in transgenic animals (+) but not wildtype littermates (-). (TIF) [file pone.0029486.s001.tif]

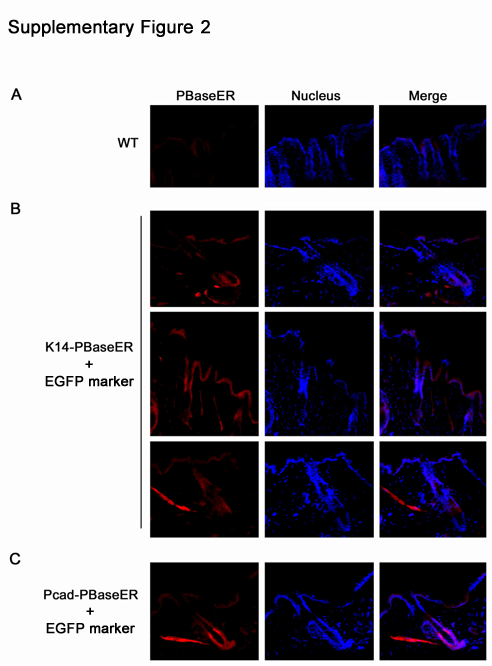

Supplement: Figure S2 — Expression of co-injected transgenes is not affected by fluorescent eye markers. Immunofluorescent staining of frozen skin sections from (A) WT, (B) K14-PBaseER (3 independent lines), and (C) Pcad-PBaseER (1 line) shows expression as expected in the epidermis and hair follicles. Note that ectopic fluorescence from the eye markers was not observed. (TIF) [file pone.0029486.s002.tif]

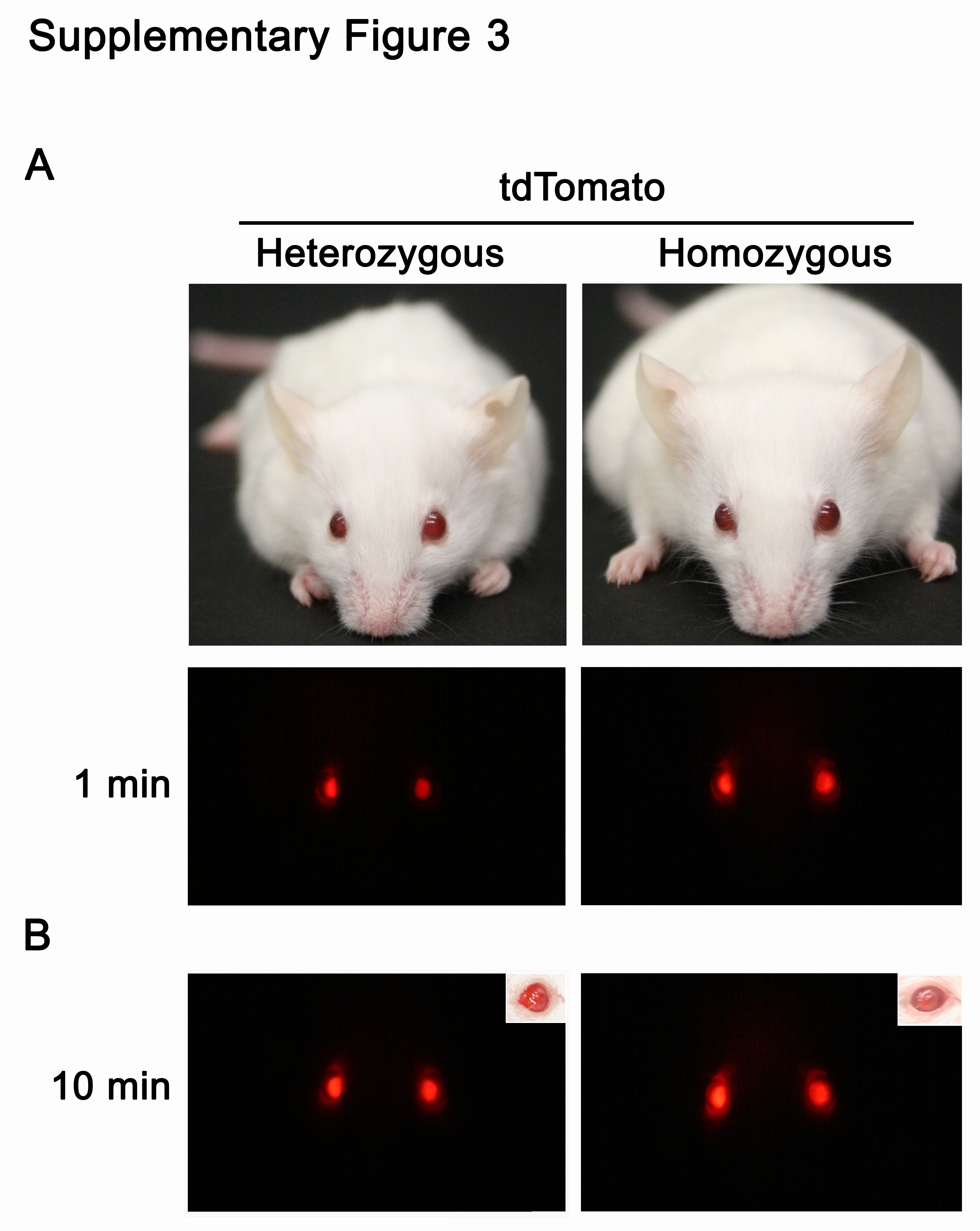

Supplement: Figure S3 — Anesthesia-induced acute cataracts affect fluorescent eye marker intensity. (A) Heterozygous and homozygous adult mice carrying tdTomato markers can be easily identified under a handheld flashlight. Mice imaged quickly have not developed cataracts. (B) tdTomato marker intensity increases following acute cataract formation (inset). Mice were imaged following ten minutes of anesthesia. (TIF) [file pone.0029486.s003.tif]
