# Supplementary material for: Characterization of Fluorescent Eye Markers for Mammalian Transgenic Studies
Source: PLoS One. 2011 Dec 28;6(12):e29486. doi: 10.1371/journal.pone.0029486 (PMC3247282; doi:10.1371/journal.pone.0029486)
Supplement: Table S1 — Fluorescent eye marker combinations. (DOC) [file pone.0029486.s004.doc]

**Table S1 Fluorescent eye marker combinations.**

|  | mCFP | EGFP | mOrange | tdTomato | mPlum |
| --- | --- | --- | --- | --- | --- |
| mCFP |  | ND | + | + | + |
| EGFP | ND |  | + | + | + |
| mOrange | + | + |  | - | + |
| tdTomato | + | + | - |  | ND |
| mPlum | + | + | + | ND |  |

+ = double transgenics can be distinguished from either single transgenic, - = single and double transgenics can not be distinguished, ND = not determined
